# Antibiotic-Resistant Bacteria in Drinking Water Across Twelve Regions of Ghana: Strengthening Evidence for National Surveillance

**DOI:** 10.3390/tropicalmed10100291

**Published:** 2025-10-14

**Authors:** Karyn Ewurama Quansah, Hawa Ahmed, Pruthu Thekkur, George Kwesi Hedidor, Lady Asantewah Boamah Adomako, Regina Ama Banu, Mark Osa Akrong, Selorm Borbor, Nawal Moro Buri, Mohammed Bello, Ebenezer Worlanyo Wallace-Dickson, Gerard Quarcoo, Emmanuel Martin Obeng Bekoe, Maria Zolfo

**Affiliations:** 1 Environmental Biology, Biotechnology and Health Division, Council for Scientific and Industrial Research-Water Research Institute (CSIR-WRI), Achimota, Accra P.O. Box AH38, Ghana; hawaahmed360@yahoo.com (H.A.); asantewa84@gmail.com (L.A.B.A.); reginamabanu@gmail.com (R.A.B.); markosaakrong@gmail.com (M.O.A.); borborviich.sb@gmail.com (S.B.); khernisha2007@yahoo.com (N.M.B.); yarbello7@gmail.com (M.B.); geroquabs@gmail.com (G.Q.); nana2g8@gmail.com (E.M.O.B.); 2 International Union Against Tuberculosis and Lung Disease (The Union), 2 Rue Lantier, 75001 Paris, France; pruthu.tk@theunion.org; 3 World Health Organization, Country Office Roman Ridge, Accra P.O. Box MB142, Ghana; khedidor@gmail.com; 4 Department of Environmental Quality and Laboratory Services, Environment Protection Authority, Accra P.O. Box M326, Ghana; worleben37@gmail.com; 5 Institute of Tropical Medicine, 2000 Antwerp, Belgium; mzolfo@itg.be

**Keywords:** antimicrobial resistance (AMR), *Escherichia coli*, multidrug resistance, One Health, operational research, *Pseudomonas aeruginosa*, Sustainable Development Goal (SDG 6), SORT IT, West Africa

## Abstract

Antimicrobial resistance (AMR) surveillance plays a critical role in tracking emerging trends and informing evidence-based policies. This study assessed bacterial contamination and resistance profiles of *Escherichia coli* and *Pseudomonas aeruginosa* in 1886 drinking water samples from 12 regions of Ghana between April 2024 and April 2025. Findings were compared to a baseline study from the Greater Accra region (2022). Water samples analysed included sachet, bottled, tap, borehole, well, and surface water. Isolates were tested for antibiotic susceptibility using the Kirby–Bauer disk diffusion method. The majority of treated and packaged water samples were free from bacterial contamination. *E. coli* was frequently detected in untreated surface water (68%) and well water (63%). *E. coli* isolates from untreated water samples exhibited high resistance to cefuroxime (74%) and amoxicillin-clavulanate (50%); resistance to gentamicin increased from 3% in 2022 to 35% in 2025, while ertapenem resistance rose from 6% to 18%. Multidrug-resistant (MDR) *E. coli* isolates were found in samples from eight regions, and MDR *P. aeruginosa* in three, mostly from borehole water. These findings highlight the urgency to integrate AMR surveillance into national water quality initiatives, along with coordinated public health interventions, to educate communities on household water treatment practices and the health risks posed by AMR.

## 1. Introduction

Access to safe drinking water is fundamental to socioeconomic development [1], which is also essential for achieving Sustainable Development Goal 6: “ensure the availability and sustainable management of water and sanitation for all” [2]. Microbial contamination of water is a serious public health threat [3], causing illnesses such as diarrhoea and enteritis, which can result in significant morbidity and mortality [4,5]. Despite global progress, an estimated 2.1 billion people worldwide still lack access to safely managed drinking water. [6]. The burden is greatest in low-and middle-income countries (LMICs), where poor water, sanitation, and hygiene (WASH) systems leave populations more vulnerable [2]. In sub-Saharan Africa, over 50% of the population relies on unsafe drinking water sources [7], which may contain drug-resistant microorganisms. This may result in the high incidence of water-borne diseases, which may be difficult to treat [8]. Hence, unsafe water contributes to the development and spread of antimicrobial resistance (AMR) [9,10,11,12,13]. In Ghana, factors including the overuse of antibiotics in livestock production [14], runoff from agricultural lands [12], and inadequate Water, Sanitation, and Hygiene (WASH) infrastructure [9,15,16], promote the spread of drug-resistant microorganisms in drinking water sources [17].

Globally, limited data exists on AMR from drinking water-associated microorganisms [10,18,19]. In Africa and in Ghana, a few studies have reported on antibiotic-resistant bacteria in drinking water sources [20,21,22,23]. Surveillance of drinking water is therefore key to tracking AMR trends and guiding targeted, evidence-based interventions [24,25,26].

Ahmed et al. [22] investigated bacterial contamination and resistance in the Greater Accra Region, identifying total coliforms (TC), total heterotrophic bacteria (THB), *Escherichia coli* (*E. coli*) and *Pseudomonas aeruginosa* (*P. aeruginosa*) from various water types. While over 50% of samples were free of *E. coli* and *P. aeruginosa*, these pathogens were detected in tap and groundwater. *E. coli* showed high resistance to cefuroxime, trimethoprim-sulfamethoxazole, and amoxicillin-clavulanate, and *P. aeruginosa* showed high resistance to aztreonam. Over half of the *E. coli* isolates were also multidrug-resistant [22].

Based on findings from the Greater Accra Region, Ahmed et al. [22] recommended nationwide AMR surveillance in drinking water [22]. To act on this recommendation, the current study analysed drinking water samples submitted for routine microbiological testing to the Council for Scientific and Industrial Research–Water Research Institute (CSIR-WRI) from twelve regions across Ghana between April 2024 and April 2025. The study assessed the presence of *E. coli*, *P. aeruginosa*, TC, and THB, as well as the antibiotic resistance profiles of *E. coli* and *P. aeruginosa*.

## 2. Materials and Methods

### 2.1. Study Design

This study used a cross-sectional design, based on routinely collected data from the water examination database at CSIR-WRI, integrating antibiotic sensitivity results of bacterial isolates from 1886 samples received for analysis between April 2024 and April 2025, from twelve regions in Ghana. Additionally, the study compared these findings with baseline data from drinking water samples submitted to CSIR-WRI from the Greater Accra region between December 2021 and March 2022, as reported by Ahmed et al. [22].

### 2.2. General Setting and Regions Included in Water Sample Surveillance

Ghana lies in West Africa and has a population of about 32.8 million [27]. The country is divided into 16 regions, and further subdivided into 260 metropolitan, municipal, and district assemblies (MMDA) for administrative purposes.

Twelve of these regions (Greater Accra, Central, Western, Ashanti, Northern, Eastern, Volta, North East, Oti, Upper East, Upper West, Ahafo) regularly submit water samples for microbiological analysis. Four regions—Greater Accra, Ashanti, Eastern, and Central account for over half (54%) of the population. Nationally, only 44.5% of the population has access to safely managed drinking water [28,29]. In the Greater Accra region, where the population is largely urban, water access is uneven. Affluent and planned residential areas typically benefit from piped water services, while low-income and peri-urban communities often lack access to the piped network and rely instead on non-piped sources such as tanker deliveries, sachet water, and boreholes [30,31]. In the Central, Eastern, Volta, and Ahafo regions, urban centres generally have access to piped water. However, peri-urban and rural populations in these areas mostly depend on groundwater sources [32]. The Western region, a business hub due to extensive mining operations and the emerging oil industry, has experienced rapid population growth, straining the piped water supply provided by the Ghana Water Company Limited, Ghana. As a result, groundwater has become a critical source of drinking water in these communities [33]. The Northern, North East, Oti, Upper West, and Upper East regions face extended and severe dry seasons, leading to significant water access challenges. Residents in these areas rely on a combination of rainwater harvesting, seasonal streams, hand-dug wells, and drilled boreholes [20,32]. In the Ashanti region, groundwater also serves as the main water source [34]. Additionally, untreated surface water, informal water sources, and sachet water are essential sources of water to many communities across the country [34,35]. This reliance may be attributed to the high cost of treated piped municipal water, which is mostly inconsistent and unreliable in supply when available [35].

The Ghana Standards Authority (GSA) is responsible for overseeing the quality assurance of drinking water. Testing laboratories also follow World Health Organisation (WHO) standards and guidelines. According to GSA standards, *E. coli*, *P. aeruginosa*, and TC should have 0 colony-forming units (CFU) per 100 mL of water tested, while THB should be below 500 CFU/mL [36]. Currently, there is no mandatory requirement to perform Antibiotic Susceptibility Testing (AST) on routinely collected drinking water samples to detect antibiotic-resistant bacteria.

### 2.3. Specific Setting

The CSIR-WRI, in Accra, Ghana, is one of the 13 institutes of the CSIR, which is the leading public research organisation in Ghana. The CSIR-WRI provides scientific data and offers strategies and services for the management and use of Ghana’s water resources, contributing to the socio-economic advancement of various sectors, particularly in health, industry, agriculture, and energy. CSIR-WRI operates two water quality testing laboratories located in Accra and Tamale that receive and analyse drinking water samples from multiple regions throughout Ghana, averaging around forty samples per week. These samples, which consist of water from boreholes, hand-dug wells, surface water, tap water, sachets, and bottled water, are submitted by water production companies or individuals looking to evaluate their water quality. The samples are classified as either raw (water from an untreated water source) or treated (water treated with some or all of the following: ultraviolet (UV) light disinfection, filtration, reverse osmosis (RO), and chlorination). Sample submission is voluntary; there is therefore a potential for sampling bias, as treated water samples may be over-represented relative to untreated community sources.

### 2.4. Baseline Study, Dissemination, and Recommendations

In October 2022, a Structured Operational Research and Training Initiative (SORT IT) module was conducted to equip researchers with the tools needed to effectively communicate the findings of the baseline study [22] to key stakeholders involved in AMR in Ghana (Table 1; SORT IT report (https://tdr.who.int/docs/librariesprovider10/sort-it/8.-ahmed_hawa.pdf?sfvrsn=79f2f586_7) accessed on 7 July, 2024). The results were presented to stakeholders from the environment, water, and sanitation sectors, as well as to the National AMR Committee.

Following these presentations, several recommendations were proposed (Appendix A), including:

(a) Continuous surveillance of drinking water sources and incorporation of AST into routine water analysis at CSIR–WRI;
(b) Immediate stakeholder engagement and community education on the risks of antibiotic-resistant pathogens in drinking water and cost-effective water treatment methods;
(c) Policy focus on increasing the number of sewage treatment plants in Accra;
(d) Enforcement of legislation to prevent indiscriminate discharge of household sewage into the environment and water bodies.

Recommendation (b) was partially implemented by the Ministry of Sanitation and Water Resources (MSWR), which used findings from the baseline study to advocate for the Water Safety Campaign and raise awareness in communities. Ghana Water Company Limited (GWCL) also continued routine maintenance and replacement of water distribution pipelines to safeguard water quality; however, this was not directly linked to the baseline study. Recommendations (c) and (d) remain unimplemented due to their high time and resource demands.

As a result, the current impact assessment study focused on recommendation (a), aiming to strengthen the evidence base for timely and effective interventions through continuous surveillance and the incorporation of AST into routine water quality monitoring.

### 2.5. Sample Collection

Borehole, well, and surface water samples were collected in 500 mL sterile bottles provided by CSIR-WRI. Samples were labelled and submitted to the lab in insulated cool boxes with ice packs at 4 °C. All samples were analysed within 24 h. Sachet and bottled water samples were received in their original packaging.

Table 1 presents the characteristics of the water samples analysed from April 2024 to April 2025.

### 2.6. Laboratory Analyses

Water samples were analysed following the procedures outlined in the Standard Methods for the Examination of Water and Wastewater [37]. The membrane filtration technique was used to detect TC, *E. coli*, and *P. aeruginosa*. 100 mL was filtered and cultured on selective media: Chromocult Coliform Agar (Merck Millipore) for TC and *E. coli*, and Cetrimide Agar (Oxoid) for *P. aeruginosa*. Plates were incubated at 37 ± 2 °C for 24 h. Bacterial colonies were counted using a colony counter. Total coliform bacteria appeared as salmon red-coloured colonies, and *E. coli* as dark blue to violet-coloured colonies. *P. aeruginosa* appeared as yellow-green to blue-coloured colonies with irregular margins on cetrimide agar. Cetrimide plates were examined under ultraviolet light to detect the presence of fluorescein.

Total heterotrophic bacteria were enumerated using the pour plate method on nutrient agar, with plates incubated at 37 ± 2 °C for 48 h. Microbial counts were reported as colony-forming units (CFU) per 100 mL for TC, *E. coli*, and *P. aeruginosa*, and per mL for THB. From each positive sample, one to five presumptive colonies of *E. coli* and *P. aeruginosa* were randomly selected. If a plate had fewer than 5 CFU/100 mL, all colonies were chosen. For plates with more than 5 CFU/100 mL, a maximum of five colonies were selected. All selected colonies were then sub-cultured for purification and confirmed using Matrix-Assisted Laser Desorption Ionization–Time of Flight Mass Spectrometry (MALDI-TOF MS; Bruker MALDI Biotyper, Billerica, MA, USA).

### 2.7. Antibiotic Sensitivity Testing

Pure isolates from each sample were randomly selected for antibiotic sensitivity testing using the Kirby–Bauer Disc Diffusion method on Mueller-Hinton agar as recommended by Clinical Laboratory Standards Institute (CLSI) 2025 guidelines [38]. Zones of inhibition were measured in millimetres and recorded for each antibiotic.

Eleven antibiotics representing different classes of antibiotics were used. These antibiotics were selected because they are commonly prescribed in the management of infections in Ghana [39]. The selection was also made to ensure comparability with the baseline study, as the antibiotics chosen were the same as those used in the baseline study. In the current study, however, amikacin was used in place of gentamicin in the baseline study, as gentamicin is currently not an antibiotic recommended for antibiotic sensitivity testing of *P. aeruginosa* isolates under the CLSI 2025 guidelines [38].

Nine antibiotics, ertapenem (10 µg), gentamicin (10 µg), ciprofloxacin (5 µg), aztreonam (30 µg), trimethoprim–sulphamethoxazole 1.25/23.75 µg), amoxicillin-clavulanate (20/10 µg), chloramphenicol (30 µg), cefuroxime (30 µg) and ceftriaxone (30 µg) were tested against *E. coli* isolates.

Amikacin (30 µg), ciprofloxacin (5 µg), aztreonam (30 µg), and piperacillin–tazobactam (100/10 µg) were also used for *P. aeruginosa* isolates. Multi-drug resistance (MDR) of the isolates, that is, resistance to three or more classes of antibiotics, was determined according to Magiorakos, 2012 [40].

### 2.8. Quality Control Procedures

Negative controls included plating 100 mL of sterile distilled water and incubating concurrently with the tested samples. This was performed to ensure that bacterial loads recovered from samples were not influenced by laboratory conditions. Reference organisms *P. aeruginosa* ATCC 27853 and *E. coli* ATCC 25922 were used as controls following CLSI guidelines to ensure that the antibiotic disc diffusion process was consistent.

### 2.9. Data Collection and Validation

Information on samples, sample source locations, bacterial loads, and resistance profiles was entered into the CSIR-WRI electronic database by laboratory technical officers. The variables of interest for this study were extracted from the laboratory records and entered into EpiCollect5 v5.1.5.2 (Oxford University, Centre for Genomic Pathogen Surveillance) mobile application by trained data assistants responsible for data entry. Data was then extracted into an electronic data file (Microsoft Excel), which was stored on a password-protected computer in the laboratory. To ensure data validation, all data in the Excel file was cross-checked with the raw data in the laboratory register by the principal investigator. Samples with missing or incomplete data were excluded from the specific analyses concerned, but retained in analyses where complete information was available; no data imputation was performed.

### 2.10. Data Analysis and Statistics

Data was imported and analysed using Jamovi version 2.3.28. Descriptive statistics, including frequencies and proportions, were used to summarise the characteristics of water samples from all twelve (12) regions. Non-parametric statistics were applied to the data in line with the data distribution.

Chi-squared tests were used to compare water sample characteristics from the Greater Accra Region between the baseline study (December 2021–March 2022) and the current study (April 2024–April 2025).

The presence of *E. coli*, *P. aeruginosa*, THB and TC was summarised by sample type (borehole, surface, well, tap, sachet, and bottled water) using frequencies and proportions. These microbial indicators from the Greater Accra Region were also compared between the baseline study and the current study using the Chi-squared test.

The median and interquartile range (IQR) were used to summarise the bacterial loads of *E. coli*, *P. aeruginosa* THB and TC in the water samples stratified by the sample type. This was compared to Ghana Standard and WHO guidelines for drinking water of 0 CFU/mL for all bacteria analysed [36,41] and <500 CFU/mL for THB [36].

The antibiotic resistance profiles and MDR of the *E. coli* and *P. aeruginosa* among all water samples were summarised using frequencies and percentages. Comparisons across treatment status (treated vs. untreated water) were conducted using the Chi-squared test. Similarly, resistance profiles and MDR status of isolates from the Greater Accra Region were compared between the baseline study period (December 2021–March 2022) and the current study period (April 2024–April 2025) using the Chi-squared test. A significance level of *p* ≤ 0.05 was applied.

The geographic distribution of water sources and MDR patterns was described by mapping GPS coordinates using ArcGIS Pro 3.4.0 software.

## 3. Results

### 3.1. Sample Characteristics of the Current Study

A total of 1886 potable water samples were received and analysed at the CSIR-WRI Microbiology Laboratory from twelve regions of Ghana (Greater Accra, Central, Western, Ashanti, Northern, Eastern, Volta, North East, Oti, Upper East, Upper West, Ahafo) between April 2024 and April 2025. Most samples came from the Greater Accra Region (1325; 70.2%), followed by the Eastern (151; 8.0%), Central (125; 6.6%), and Western (105; 5.6%) regions (Table 1).

Treated water (sachet, bottled, tap, borehole, and well) constituted 84.5% of all samples, while untreated sources (surface, borehole, and well water) accounted for 15.5% (*n* = 293) of all samples (Table 1).

Overall, coliforms and *P. aeuriginosa* were not detected in 82% (1555/1886) of samples. All bottled water samples were free of *E. coli.* Surface water was most contaminated, with 66.7% (8/12) of samples positive for *E. coli.* Well water also showed high contamination (62.5%; 40/64), followed by borehole (11.8%; 79/667), tap (3.3%; 9/274), and sachet water (1.0%; 7/699).

*P. aeruginosa* contamination varied: tap water had the highest counts (144 CFU/mL; IQR 62–279), followed by borehole water (60 CFU/mL; IQR 18–279). Although 21.9% of well water samples were positive for *P. aeruginosa*, the median remained 0 because more than half of the samples showed no detectable counts, reflecting a skewed distribution. Some packaged water samples (sachet and bottled) were also contaminated (93 CFU/mL; IQR 9–186, and 13 CFU/mL; IQR 8–279, respectively) (Table 2).

#### 3.1.1. Antibiotic Resistance Profiles of *E. coli* and *P. aeruginosa* During the Current Study

We tested 135 *E. coli* isolates against nine antibiotics and 217 *P. aeruginosa* isolates against four antibiotics. *E. coli* isolates showed the highest resistance to cefuroxime (80% and 74.3%) and amoxicillin-clavulanate (46.7% and 49.5%), with lower resistance to chloramphenicol (10% and 13.3%) and ciprofloxacin (10% and 24.8%), for treated and untreated water sources, respectively. No significant differences were observed between treated and untreated sources.

For *P. aeruginosa*, isolates from untreated water showed higher resistance to amikacin (24.2% vs. 12.3%, *p* = 0.022) and ciprofloxacin (14.7% vs. 5.7%, *p* = 0.026) compared to those from treated water. Resistance to aztreonam and piperacillin–tazobactam did not differ significantly between treated and untreated samples (Table 3).

#### 3.1.2. Multi-Drug Resistance Profiles of *E. coli* and *P. aeruginosa* in the Current Study

Eight of the twelve regions reported MDR *E. coli* isolates in samples submitted for analysis. The highest proportions were found in the Greater Accra (60.9%), North East (17.2%), and Ashanti (7.8%) regions. In the Greater Accra region, MDR isolates were most commonly found in borehole and well water samples; in the North East region, MDR isolates were detected in surface water samples.

**Figure 1:**
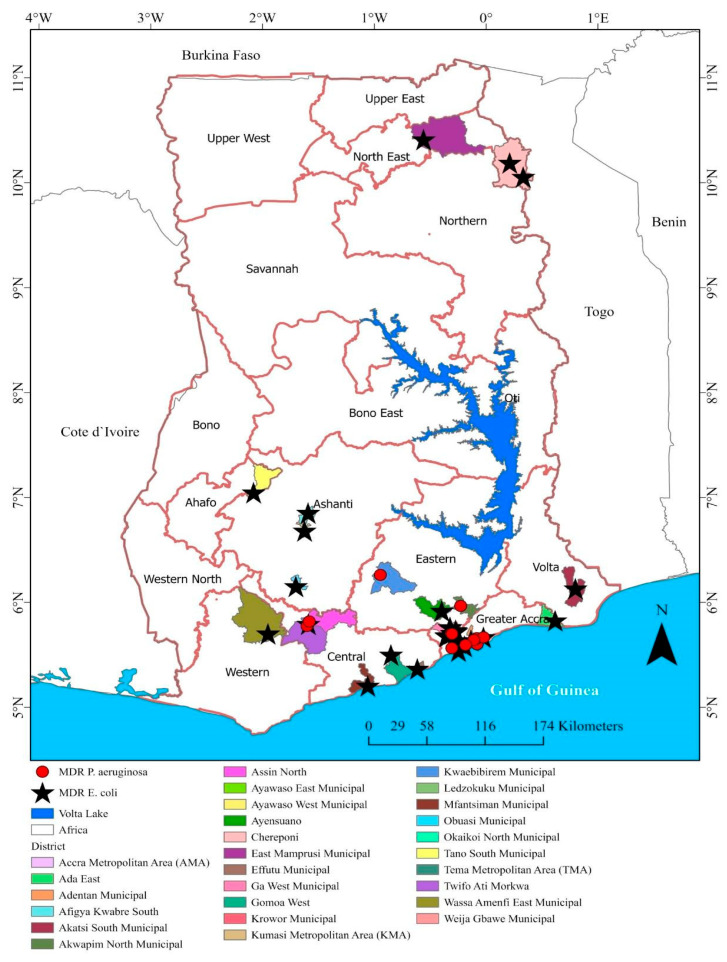
A spot map of Ghana showing source locations (districts) and MDR (resistance to ≥3 tested classes of antibiotics) *E. coli* and *P. aeruginosa* isolates from water samples for the current study (April 2024–April 2025).

MDR P. aeruginosa isolates were found in three regions: the Greater Accra region (44.4%), Central (33.3%), and Eastern (22.2%). Most MDR *P. aeruginosa* isolates were found in borehole water, particularly in the Central (60%) and Eastern (30%) regions (Figure 1).

### 3.2. Comparison of Samples in the Greater Accra Region Between the Baseline Study and the Current Study

**Figure 2:**
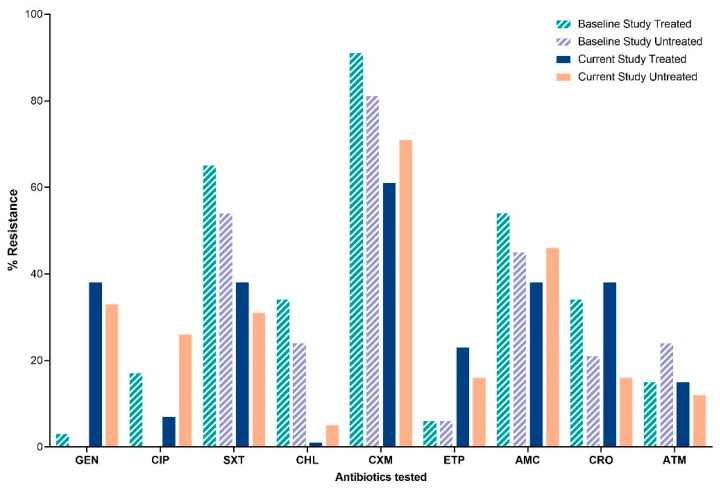
Proportions of resistant *E. coli* isolates from treated and untreated water samples from the Greater Accra region for the period (December 2021 to March 2022) compared with the baseline study period (April 2024 to April 2025). Key: GEN: gentamicin; CIP: ciprofloxacin; SXT: trimethoprim–sulfamethoxazole; CHL: chloramphenicol; CXM: cefuroxime; ETP: ertapenem; AMC: amoxicillin-clavulanate; CRO: ceftriaxone; ATM: Aztreonam; % resistance: percentage of resistance to tested antibiotics.

Differences in resistance proportions of *E. coli* isolates were observed between the baseline study (115) and the current study (84). *E. coli* isolates showed increased resistance in treated water to gentamicin (3.7% to 38.5%, *p* < 0.001) and ertapenem (6.1% to 23.1% in treated water; 6.1% to 16.9% in untreated water, *p* < 0.009). Conversely, resistance declined for trimethoprim-sulfamethoxazole (66% to 39%, *p* < 0.001) and cefuroxime (92% to 62% in treated, 82% to 72% in untreated water, *p* < 0.001) (Figure 2).

**Figure 3:**
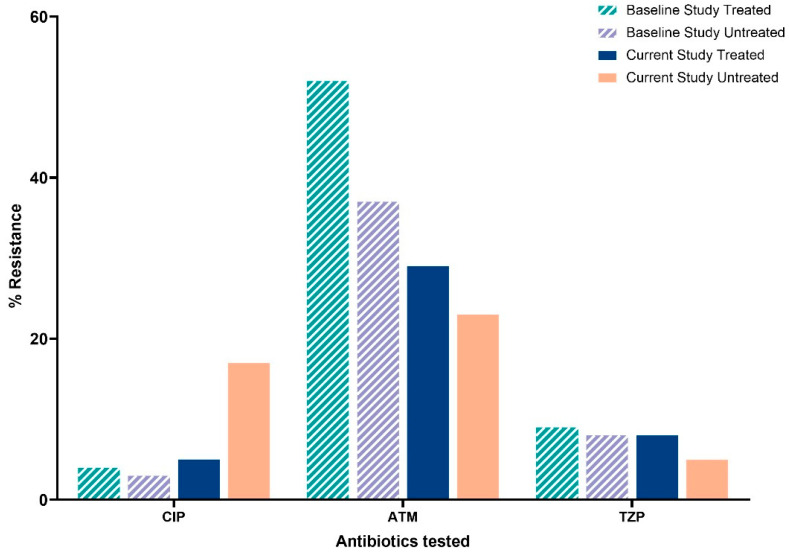
Proportions of resistant *P. aeruginosa* isolates from treated and untreated water samples from the Greater Accra region for the period (December 2021 to March 2022) compared with the baseline study period (April 2024 to April 2025). Key: CIP: ciprofloxacin; ATM: Aztreonam; TZP: piperacillin-tazobactam; % resistance: percentage of resistance to tested antibiotics.

For *P. aeruginosa*, amikacin replaced gentamicin in the current study, which prevents direct comparisons. Differences in resistance proportions of *P. aeruginosa* isolates were observed between the baseline study (202) and the current study (92). Resistance to aztreonam decreased (52.1% to 29.8%, *p* < 0.001) in treated water samples, while ciprofloxacin and piperacillin–tazobactam showed no significant changes (Figure 3).

MDR prevalence in *E. coli* isolates declined (58.3% to 46.4%, *p* = 0.050), whereas MDR in *P. aeruginosa* isolates showed no significant change (4.5% to 7.6%, *p* = 0.408) (Table 4).

## 4. Discussion

While earlier studies have explored antibiotic resistance in Ghana’s drinking water [20,21,22], this is the first study to assess the antibiotic-resistant profiles of bacteria in drinking water samples from 12 out of the 16 regions in Ghana. We identified the following key findings.

### 4.1. Microbial Water Quality

A large proportion of all samples tested (82%) were free from total coliform, *E. coli* and *P. aeruginosa* contamination, meeting WHO guidelines for safe drinking water [42]. Over 80% of treated water samples were free from *E. coli* and *P. aeruginosa* contamination. *E. coli* was not detected in any of the bottled water samples, and only 1% (7/699) of sachet water samples tested had *E. coli* contamination. These observations are similar to the 2022 baseline study, where no *E. coli* contamination was seen in packaged drinking water samples [22]. It shows that the current treatment regimens being used, similar to the baseline study (R.O., UV, filtration, chlorination), are efficient at removing or substantially reducing levels of bacterial contamination. These results are consistent with findings in South Africa that demonstrated how water treatment can reduce bacterial contamination in drinking water [43].

A significant proportion of well and surface water samples in this study had high bacterial loads. Similar findings were reported in a recent study from northern Ghana, where surface waters were also found to be heavily contaminated with bacteria [20]. Although the baseline study did not include surface water samples, it did reveal that all well water samples tested contained high levels of *E. coli*, consistent with current observations. The high bacterial loads and *E. coli*, which is an indicator of faecal contamination, could be attributed to environmental exposure, poor water management and lack of adequate sanitation, allowing these water sources to become constant reservoirs of microbial contamination [20,44].

### 4.2. Antibiotic Resistance Profiles

This study revealed notable resistance patterns among the tested bacterial isolates. Of the 135 *E. coli* isolates tested, the highest proportions were resistant to cefuroxime (75.6%) and amoxicillin-clavulanate (48.9%). These high resistance trends were observed regardless of whether the water had been treated or not. Previous studies [20,45], including the baseline study [22], have similarly reported high levels of *E. coli* resistance to cefuroxime and amoxicillin-clavulanate. There was, however, a significant decline in resistance to cefuroxime, from 88.7% in the baseline study in 2022 to 70.2% in the current study in 2025, for *E. coli* isolates from the Greater Accra region. In Ghana, cefuroxime and amoxicillin-clavulanate remain among the most frequently prescribed antibiotics for clinical care; this may account for the high prevalence of bacterial resistance to these drugs [46,47,48]. Unlike previous studies from Ghana [20,22], *E. coli* isolates in the current study showed a 41.5% resistance to gentamicin, comparable to a 57.7% resistance reported in drinking water from Sudan [49]. Comparing isolates from the Greater Accra region, we noticed a significant increase in gentamicin resistance in *E. coli* from 2.6% (3/115) in the baseline study to 34.5% (29/84) in the current study (*p* < 0.001). Recent studies from pig farms [14,50] and hospital wastewater [44] in Ghana have shown that environmental exposure through the agricultural and veterinary use of aminoglycosides [14], poor sanitation [10] and widespread informal antibiotic access and use [20] may aid horizontal gene transfer among co-existing resistance genes in environmental reservoirs and may account for the high levels of gentamicin resistance [16,44,51]. These findings emphasise the complex ecological drivers of AMR beyond clinical misuse, reinforcing the need for environmental antibiotic stewardship [23].

We also observed that *E. coli* resistance to the carbapenem tested, ertapenem, increased markedly from the baseline study, 6.1% (7/115) in 2022 to 17.9% (15/84) in 2025. This pattern is similar to work performed in Sudan, where 33% of *E. coli* isolates in drinking water were resistant to imipenem. *E. coli* isolates from clinical sources in Ghana also recorded the highest levels of resistance to ertapenem (22% and 100%, respectively) in a recent study in Accra [39,52]. Despite the variations in resistance patterns, the studies suggest that carbapenem resistance is slowly emerging in Ghana [52]. The emergence of ertapenem resistance among *E. coli* isolates in drinking water in Ghana is concerning and likely driven by multiple converging factors. Firstly, the infiltration of hospital and pharmaceutical effluents into municipal or groundwater sources without adequate treatment can introduce carbapenem-resistant organisms and resistance genes into environmental reservoirs [16]. Horizontal gene transfer mediated by mobile genetic elements in aquatic ecosystems may also accelerate the dissemination of carbapenemase genes which have been detected in clinical Ghanaian isolates [16,39]. This emerging resistance is of serious concern, as ertapenem is part of the ‘Watch’ group of the WHO AWaRe classification of antibiotics [53]. These antibiotics are the last resort antibiotic treatment for many Gram-negative bacteria [54]. In Ghana, carbapenems are the preferred antibiotics for managing severe bacterial infections, [39] and increased resistance in isolates from drinking water sources indicates a potential public health risk.

It also confirms continental trends reported by Olaitan et al. [55] and Osisiogu et al. [56], who highlighted a disturbing rise in resistance to last-line antibiotics across sub-Saharan Africa.

A total of 217 *P. aeruginosa* isolates were tested in this current study. Among all *P. aeruginosa* isolates, resistance was primarily observed to aztreonam (41%), while susceptibility to all other antibiotics tested remained high. *P. aeruginosa* isolates from the Greater Accra region showed a 27.2% (25/92) resistance to aztreonam. This was higher than the 18% level of resistance recorded from a similar study in Lebanon [57] but lower than 48% (97/202) in 2022 from the baseline study.

### 4.3. Multidrug Resistance

Resistance profiling of isolates revealed high levels of resistance to multiple antibiotics, with multidrug-resistant strains of both species found across nearly all water sample types. Similar to the baseline study, most MDR isolates were from untreated water sources. MDR *E. coli* isolates were detected in samples from eight out of the twelve regions, while MDR *P. aeruginosa* was identified in samples from three regions. The Greater Accra region recorded the highest proportion of MDR isolates. However, this region also accounted for the majority of samples received (70.2%), which may have influenced the higher number of MDR detections observed.

The sustained detection of MDR isolates in both treated and untreated water types supports the claim that antibiotic-resistant bacteria may now be increasingly present in many aquatic ecosystems [17,58].

According to Ahmed et al. [22] and Duarte et al. [59], conventional water treatment methods, such as chlorination alone, do not often eliminate antibiotic-resistant bacteria. A reduction in pipe-borne water quality occurs along distribution lines due to the formation of biofilms. The role of biofilms in the development of MDR is shown in previous studies [59], where higher levels of ARB were observed in tap water compared to water directly from the treatment plant, indicating regrowth of bacteria can occur within drinking water distribution systems. This may therefore likely be the reason for the presence of antibiotic-resistant isolates found in treated water samples. These findings provide a broader picture of the geographical spread of MDR in Ghana’s drinking water sources.

### 4.4. Strengths and Limitations

The wider coverage of samples in this study helped to identify possible AMR hotspots that may not have been detected in smaller or single-region studies. The study also analysed a variety of water sources, including borehole, sachet, bottled, tap, well, and surface water, which allowed for a more detailed understanding of contamination and resistance patterns across different water sample types. Additionally, the research focuses on a national priority area, which is antimicrobial resistance in the environment. The antibiotics tested were selected based on the WHO AWaRe classification, covering both Access, Watch and Reserve groups [53], which are important for public health. The conduct and reporting of the study also followed the STROBE guidelines for observational research, which added to its scientific reliability.

This study, however, has some limitations. It relied on samples that were received for analysis at the lab during the study period. This may have introduced some sampling bias and treated water samples being over-represented. A large number of samples were from the Greater Accra region, and fewer samples were from the other regions. This makes it difficult to compare resistance patterns between regions. Laboratory-based surveillance may therefore not reflect contamination at the household/community level. The study also did not employ a seasonal breakdown of the samples received, so inference of antibiotic resistance by seasons could not be made. Lastly, as the study did not include genomic sequencing, it is not possible to identify the specific resistance genes or understand how resistance may be spreading.

### 4.5. Public Health Implications and Recommendations

Well and surface waters continue to harbour high levels of bacterial contamination and resistant organisms, suggesting that current sanitation, wastewater management, and rural water safety interventions are insufficient. The data indicates that the majority of treated and packaged drinking water samples, which usually undergo multiple treatment methods, are safe from bacterial contamination and thus free from antibiotic-resistant isolates. However, the high gentamicin and ertapenem resistance rates in *E. coli* isolates tested suggest that empirical treatment protocols in Ghana may need revision and imply that these antibiotics may no longer work as effective first-line and last resort treatment for *E. coli* infections without susceptibility tests. Compared with the baseline study [16], these findings offer new insight into the evolving resistance patterns and highlight the need to integrate AST into routine water quality monitoring. The results have clear public health relevance, emphasising the urgency of continued surveillance and the adoption of One Health strategies to address environmental AMR risks.

Considering the changing trends in AMR and the detection of MDR bacteria in different locations across the country, it is recommended that future surveillance must be proactive, nationwide, and genomically informed. This would provide a deeper insight into the genetic mechanisms of resistance and allow for the identification of resistance genes, track transmission pathways, pinpoint environmental reservoirs and potential sources of contamination. Special focus should be placed on high-risk organisms such as Extended-Spectrum Beta-Lactamase (ESBL) and carbapenemase-producing bacteria, which pose serious public health threats due to limited treatment options, making critical antibiotics ineffective.

Immediate action is needed to engage stakeholders and educate communities about the health risks posed by antibiotic-resistant bacteria in drinking water. Public awareness campaigns should also promote affordable, effective household water treatment methods. At the policy level, there is a need to strengthen water safety management as the most immediate and actionable public health priority. Routine monitoring, improved disinfection, and enforcement of water quality standards are crucial for protecting populations from exposure to resistant pathogens through contaminated drinking water. Broader antimicrobial stewardship measures, including the rational use of antibiotics in both human and veterinary medicine, are crucial for reducing the environmental impact of antimicrobial resistance. There must also be a focused effort to expand sewage treatment infrastructure, especially in Accra, where existing facilities are insufficient. Without immediate, coordinated policy action, Ghana risks facing a growing wave of hard-to-treat infections stemming from its own water supply.

## 5. Conclusions

This study provides the most extensive assessment to date of microbial contamination and AMR in drinking water sources across twelve regions in Ghana. The continued presence of *E. coli* in untreated water sources, along with newly identified MDR hotspots of *E. coli* and *P. aeruginosa* across the country, and the rising resistance to WHO ‘Watch’ category antibiotics such as gentamicin and ertapenem, underscore that AMR remains a significant public health threat linked to environmental reservoirs. The findings from this study provide strong evidence to incorporate AST into routine water quality monitoring. They also highlight the importance of targeted active surveillance supported by whole-genome sequencing to better track and understand the spread of antimicrobial resistance.

## Figures and Tables

**Table 1:** Characteristics of the water samples brought to CSIR-WRI for microbial water quality analyses from twelve regions in Ghana from April 2024 to April 2025.

Characteristics	N	(%)
Total	1886	100
**Region**		
Ahafo	2	(0.1)
Ashanti	53	(2.8)
Central	125	(6.6)
Eastern	151	(8.0)
Greater Accra	1325	(70.2)
North East	12	(0.6)
Northern	30	(1.6)
Oti	18	(1.0)
Upper East	6	(0.3)
Upper West	6	(0.3)
Volta	53	(2.8)
Western	105	(5.6)
**Sample provider**		
Individual	240	(12.7)
Water company	1646	(87.3)
**Sample type**		
Borehole water	667	(35.4)
Bottled water	170	(9.0)
Sachet water	699	(37.1)
Surface water	12	(0.6)
Tap water	274	(14.5)
Well water	64	(3.4)
**Treatment methods**		
None	293	(15.5)
Chlorination only	153	(8.1)
Filtration	22	(1.2)
Reverse Osmosis	2	(0.1)
Ultraviolet	2	(0.1)
Reverse Osmosis (RO) and Ultraviolet (UV)	22	(1.2)
Chlorination and filtration	242	(12.8)
R.O., UV, Filtration, Chlorination	948	(50.3)
Sand, Carbon, R.O., Filtration, UV	202	(10.7)

N: Total number of samples; %: percentage.

**Table 2:** Bacterial load (cfu/100 mL) stratified by type of the sample brought to CSIR-WRI for microbial water quality analyses from twelve regions in Ghana from April 2024 to April 2025.

		Type of Bacteria							
Sample Type	N	Total Coliforms		*E. coli*		*P. aeruginosa*		THB	
		n (%)	M (IQR)	n (%)	M (IQR)	n (%)	M (IQR)	n (%)	M (IQR)
Sachet	699	8 (1.1)	139.5 (12.3–604.5)	7 (1.0)	10.0 (4.0–13.5)	23 (3.3)	9.0 (9.0–186.0)	426 (60.9)	9.0 (2.0–247.0)
Bottle	170	3 (1.8)	1.0 (1.0–93.5)	-	-	5 (2.9)	13.0 (8.0–279.0)	105 (61.8)	9.0 (2.0–520.0)
Borehole	667	222 (33.3)	372.0 (76.3–930.0)	79 (11.8)	11.0 (4.5–60.0)	36 (5.4)	60.0 (18.0–279.0)	574 (86.1)	936.0 (10.0–3276.0)
Tap	274	35 (12.8)	180.0 (28.0–651.0)	9 (3.3)	4.0 (2.0–14.8)	11 (4.0)	144.0 (62.0–279.0)	212 (77.4)	18.0 (3.0–1012.0)
Well	64	51 (79.7)	1116.0 (465.0–2325.0)	40 (62.5)	40.0 (10.0–92.0)	14 (21.9)	0 (0.0–35.5)	62 (96.9)	2808.0 (702.0–3744.0)
Surface water	12	12 (100)	1441.50 (866.3–2409.5)	8 (66.7)	40.0 (10.0–92.0)	3 (25)	2.0 (0.0–2.0)	12 (100)	3978.0 (3744.0–4329.0)

cfu: colony forming units; M: Median; IQR: Interquartile range; N: number of samples tested; n: number of contaminated samples; %: percentage of contaminated samples; THB: total heterotrophic bacteria.

**Table 3:** Number and proportion of antibiotic-resistant *Escherichia coli* and *Pseudomonas aeruginosa* isolates in drinking water samples (stratified by treatment status) brought to CSIR-WRI for analyses from twelve regions in Ghana from April 2024 to April 2025.

	*E. coli*				*P. aeruginosa*			
Antibiotics (μg)	Total (N = 135)	Treated (N = 30)	Untreated (N = 105)	*p* ^a^	Total (N = 217)	Treated (N = 122)	Untreated (N = 95)	*p* ^a^
	n (%)	n (%)	n (%)		n (%)	n (%)	n (%)	
Amoxicillin-clavulanate (20/10 µg)	66 (48.9)	14 (46.7)	52 (49.5)	0.782	-	-	-	-
Gentamicin (10 µg)	56 (41.5)	13 (41.0)	43(43.3)	0.815	-	-	-	-
Ciprofloxacin (5 µg)	29 (21.5)	3 (10.0)	26 (24.8)	0.083	21 (9.7)	7 (5.7)	14 (14.7)	0.026
Aztreonam (30 µg)	17 (12.6)	4 (13.3)	13 (12.4)	0.890	89 (41)	49 (40.2)	40 (42.1)	0.773
Cefuroxime (30 µg)	102 (75.6)	24 (80.0)	78 (74.3)	0.521	-	-	-	-
Ertapenem (10 µg)	21 (114)	4 (13.3)	17 (16.2)	0.703	-	-	-	-
Trimethoprim-sulfamethoxazole (1.25/23.75 µg)	41 (30.4)	7 (23.3)	34 (32.4)	0.342	-	-	-	-
Chloramphenicol (30 µg)	17 (12.6)	3 (10.0)	14 (13.3)	0.627	-	-	-	-
Ceftriaxone (30 µg)	24 (25.2)	6 (20.0)	18 (17.1)	0.718	-	-	-	-
Piperacillin-tazobactam (100/10 µg)	-	-	-	-	14 (6.5)	10 (8.2)	4 (4.2)	0.236
Amikacin (30 µg)	-	-	-	-	38 (17.5)	15 (12.3)	23 (24.2)	0.022

N: number of total isolates tested; n: number of resistant isolates; “-”: antibiotic was not tested against isolates; *p* ^a^: *p* value (*p* < 0.05) comparing treated and untreated *E. coli* and *P. aeruginosa* isolates using the Chi-square test.

**Table 4:** Number and proportion of multidrug resistance in *E. coli* and *P. aeruginosa* isolates in drinking water samples tested from the Greater Accra region, Ghana, stratified by sample type for the baseline study (December 2021 to March 2022) and the current study (April 2024 to April 2025).

	*E. coli*					*P. aeruginosa*				
	Baseline study		Current study			Baseline study		Current study		
	n (N)	%	n (N)	%	*p* value ^a^	n (N)	%	n (N)	%	***p* value ^b^**
**Total Isolates**	67 (115)	58.3	39 (84)	46.4	0.050	9 (202)	4.5	7 (92)	7.6	**0.408**
**Sachet**	-	-	1 (5)	20	-	2 (45)	4.4	2 (27)	7.4	**0.631**
**Bottled**	-	-	-	-	-	0 (3)	0.0	1 (4)	25	**0.200 ^c^**
**Tap**	51 (79)	64.6	1 (2)	50.0	0.018 ^c^	3 (90)	3.3	1 (18)	5.5	**0.409**
**Borehole**	8 (11)	72.7	17 (37)	45.9	0.103	4 (58)	6.9	1 (33)	3.0	**0.200**
**Well**	8 (25)	32.0	20 (40)	50.0	0.092	0 (6)	0.0	3 (10)	30.0	**0.087**

n: number of multidrug resistant (MDR) isolates; N: number of isolates tested; %: percentage of MDR isolates; -: no bacteria was isolated for that sample; *p* value ^a^: comparison of MDR *E. coli* using the Chi-square test; *p* value ^b^: comparison of MDR *P. aeruginosa* using the Chi-square test; ^c^: Fisher’s Exact Test was used.

**Figure A1:**
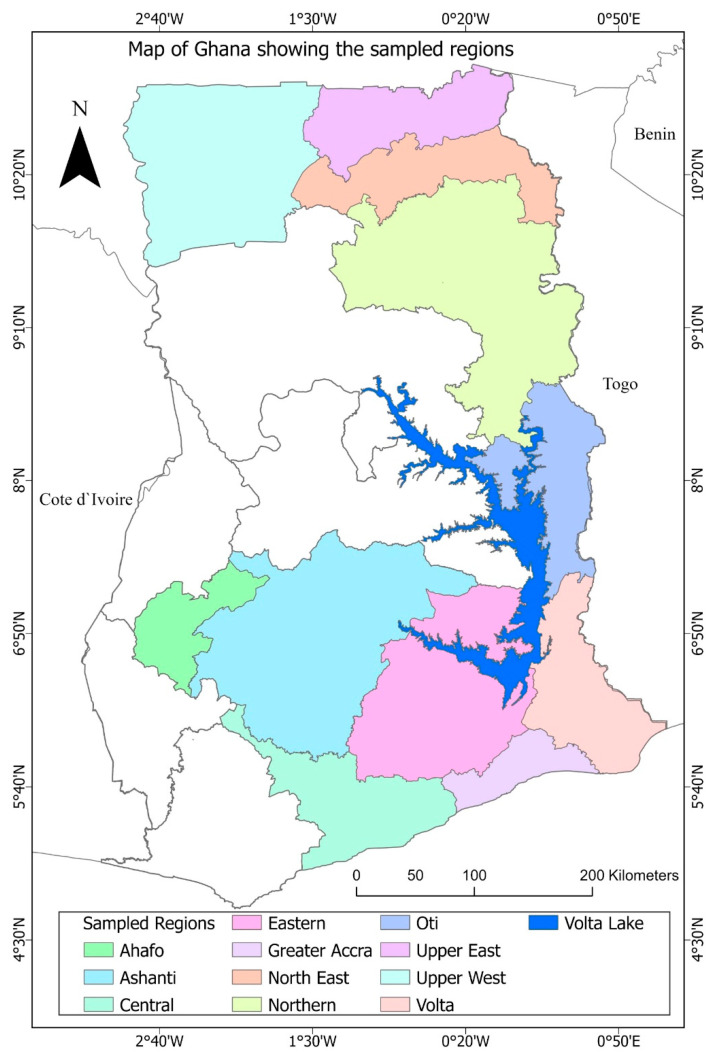
A map of Ghana showing regions from which water samples were received for the current study (April 2024–April 2025).

## Data Availability

Requests to access these data should be sent to the corresponding author.

## Abbreviations

The following abbreviations are used in this manuscript:

AMC	Amoxicillin-clavulanate
AK	Amikacin
AMR	Antimicrobial Resistance
ARB	Antibiotic-Resistant Bacteria
ATM	Aztreonam
CFU	Colony-Forming Units
CHL	Chloramphenicol
CIP	Ciprofloxacin
CLSI	Clinical Laboratory Standards Institute
CSIR	Council for Scientific and Industrial Research
CSIR-WRI	Council for Scientific and Industrial Research—Water Research Institute
CRO	Ceftriaxone
CXM	Cefuroxime
ESBL	Extended-Spectrum Beta-Lactamase
ETP	Ertapenem
FDA	Food and Drugs Authority
GNAT	Ghana National Association of Teachers
GEN	Gentamicin
GSA	Ghana Standards Authority
GWCL	Ghana Water Company Ltd./Ghana Water Company Limited
LMICs	Lower- and Middle-Income Countries
MALDI-TOF MS	Matrix-Assisted Laser Desorption Ionisation–Time of Flight Mass Spectrometry
MDR	Multidrug Resistance
MESTI	Ministry of Environment, Science, Technology and Innovation
MLG	Ministry of Local Government
MMDA	Metropolitan, Municipal, and District Assemblies
MOH	Ministry of Health
MSWR	Ministry of Sanitation and Water Resources/The Ministry of Sanitation and Water Resources
NGOs	Non-Governmental Organisations
PURC	Public Utilities Regulatory Commission
RO	Reverse Osmosis
SORT IT	Structured Operational Research Training Initiative
SXT	Trimethoprim–sulfamethoxazole
TDR	The Special Programme for Research and Training in Tropical Diseases
TC	Total Coliforms
THB	Total Heterotrophic Bacteria
TZP	Piperacillin-tazobactam
UV	Ultraviolet
WASH	Water, Sanitation and Hygiene
WHO	World Health Organisation

## Appendix A

**Table A1:** Dissemination details of the baseline operational research study conducted by Ahmed and colleagues [22], Ghana.

How	To Whom	Where (Attending People: Number)	When
Stakeholder mapping and communication planning	Study team	SORT IT Module 4 *	October 2022
Publication in a peer-reviewed journal	Researchers, AMR advocates, national and international stakeholders/general public	Journal website, email exchange Social platforms—WhatsApp, Research Gate, and LinkedIn (>100)	September 2022
Policy Brief handouts	CSIR-Water Research Institute, Researchers, AMR stakeholders	CSIR-Water Research Institute front desk, MSWR	July–September 2023
Publication uploaded to websites	National and international stakeholders/general public	Journal website (citations 5; views: 2968) TDR website	September 2022
Poster presentations and discussions	FDA, scientists, academia, research institutions, MESTI, MOH	FDA Ghana Scientific Forum (>50)	September 2023
Lightning presentations and discussions	SORT IT Ghana Cohort and relevant stakeholders from research and various government ministries	SORT IT Module 4 (30)	October 2022
Ten-minute technical PowerPoint presentation and discussions	Stakeholders from MESTI, MOH, MSWR, MLG, research institutions, academia, and media outlets	National SORT IT dissemination meeting (36)	July 2023
	National Technical Working Group on Water	Coconut Grove Hotel, Accra (30)	August 2023
	Water Safety Campaign Stakeholders	Coconut Grove Hotel, Accra (40)	September 2023
	National Co-ordinating Committee on Drinking Water Quality Management in Ghana	PURC Conference Room, GNAT Heights, Accra (20)	October 2023

Abbreviations: SORT IT—Structured Operational Research Training Initiative; AMR—Antimicrobial Resistance; CSIR—Council for Scientific and Industrial Research; MSWR—Ministry of Sanitation and Water Resources; TDR—the Special Programme for Research and Training in Tropical Diseases; FDA—Food and Drugs Authority; MESTI—Ministry of Environment, Science, Technology and Innovation; MOH—Ministry of Health; MLG—Ministry of Local Government; PURC—Public Utilities Regulatory Commission; GNAT—Ghana National Association of Teachers. * https://tdr.who.int/docs/librariesprovider10/sort-it/8.-ahmed_hawa.pdf?sfvrsn=79f2f586_ (accessed on 10 August 2024).

**Table A2:** Recommendations, action status, and action details from the baseline study by Ahmed and colleagues [22], Ghana.

Recommendation	Action Status	Details of Action (When and What)
Organise awareness programs to educate communities on additional cost-effective water-treatment methods as an immediate control measure	Implemented	October 2023 MSWR used study findings to create awareness in various communities
Inform and engage stakeholders to improve their awareness of the presence of pathogens in drinking water sources	Implemented	July 2023 National SORT IT dissemination meeting held with stakeholders from MESTI, MOH, academia, research institutions, and media
Expand environmental AMR surveillance to include drinking water sources in the national AMR action plan	Partially implemented	The National Action Plan has been revised to include drinking water in the Environmental AMR surveillance (in a draft)
Continuous surveillance of drinking water sources and the inclusion of AST as a routine parameter for water sample analysis at the CSIR–WRI	Not yet implemented	Research findings have been communicated to the management of CSIR-WRI and the Head of the Environmental Biology, Biotechnology, and Health Division
Conduct further research that investigates a larger geographical area	Ongoing	SORT IT impact assessment study includes multiple regions in Ghana
Conduct further research that identifies sources of bacterial contamination, assesses AMR risk and transmission levels	Not yet implemented	
Regular repairs, maintenance, and replacement of existing water distribution pipelines to maintain the quality of water reaching households	Ongoing	July 2023 Research findings have been disseminated to stakeholders, including GWCL, PURC, MWSR, NGOs

Abbreviations: MSWR—Ministry of Sanitation and Water Resources; SORT IT—Structured Operational Research Training Initiative; MESTI—Ministry of Environment, Science, Technology and Innovation; MOH—Ministry of Health; AMR—Antimicrobial Resistance; GWCL—Ghana Water Company Ltd.; PURC—Public Utilities Regulatory Commission; NGOs—Non-Governmental Organisations.
